# 4-Chloro-*N*-[4-(diethyl­amino)benzyl­idene]aniline

**DOI:** 10.1107/S160053681000125X

**Published:** 2010-01-16

**Authors:** Fu-Gong Zhang

**Affiliations:** a Weifang University, Weifang 261061, People’s Republic of China

## Abstract

The asymmetric unit of the title compound, C_17_H_19_ClN_2_, contains two independent mol­ecules which differ by a 180° flip in the orientation of the 4-chloro­aniline unit with respect to the diethyl­amino­benzyl­idene unit [N=C—C—C = 10.0 (3) and −170.6 (2)°]. The dihedral angles between the two aromatic rings are 64.0 (1) and 66.5 (1)° in the two independent mol­ecules.

## Related literature

For general background to Schiff base compounds in coordin­ation chemistry, see: Yu *et al.* (2007 ▶). For a related structure, see: You *et al.* (2004 ▶).
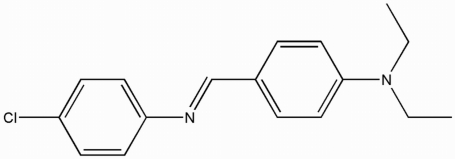


## Experimental

### 

#### Crystal data

C_17_H_19_ClN_2_
*M* *_r_* = 286.79
Monoclinic, 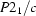

*a* = 20.153 (2) Å
*b* = 8.7434 (7) Å
*c* = 20.1446 (19) Å
β = 118.444 (2)°
*V* = 3121.0 (5) Å^3^
*Z* = 8
Mo *K*α radiation
μ = 0.24 mm^−1^
*T* = 293 K
0.25 × 0.22 × 0.18 mm

#### Data collection

Bruker SMART CCD area-detector diffractometer
Absorption correction: multi-scan (*SADABS*; Sheldrick, 1996 ▶) *T*_min_ = 0.942, *T*_max_ = 0.958
22416 measured reflections
5494 independent reflections
4266 reflections with *I* > 2σ(*I*)
*R*_int_ = 0.034

#### Refinement

*R*[*F* ^2^ > 2σ(*F* ^2^)] = 0.045
*wR*(*F* ^2^) = 0.127
*S* = 1.07
5494 reflections
361 parameters
H-atom parameters constrained
Δρ_max_ = 0.25 e Å^−3^
Δρ_min_ = −0.34 e Å^−3^

### 

Data collection: *SMART* (Bruker, 1997 ▶); cell refinement: *SAINT* (Bruker, 1997 ▶); data reduction: *SAINT*; program(s) used to solve structure: *SHELXS97* (Sheldrick, 2008 ▶); program(s) used to refine structure: *SHELXL97* (Sheldrick, 2008 ▶); molecular graphics: *SHELXTL* (Sheldrick, 2008 ▶); software used to prepare material for publication: *SHELXTL*.

## Supplementary Material

Crystal structure: contains datablocks global, I. DOI: 10.1107/S160053681000125X/ci2995sup1.cif

Structure factors: contains datablocks I. DOI: 10.1107/S160053681000125X/ci2995Isup2.hkl

Additional supplementary materials:  crystallographic information; 3D view; checkCIF report

## Figures and Tables

**Fig. 1.**
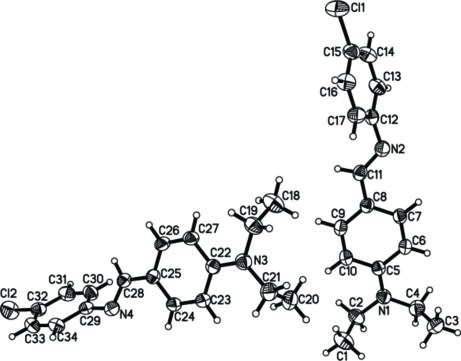
The two independent molecules of the title compound. Displacement ellipsoids are drawn at the 30% probability level.

## supplementary crystallographic information

### Comment

Schiff base compounds have been used as fine chemicals and medical substrates. They are important ligands in coordination chemistry due to their ease of preparation and can both electronically and sterically modified (Yu *et al.*, 2007). In this paper, the crystal structure of the title compound is reported.

The asymmetric unit of the title compound consists of two independent molecules, as illustrated in Fig. 1. The two molecules differ by a 180° flip in the orientation of the 4-chloroaniline unit with respect to the diethylaminobenzylidene moiety. The N4—C28—C25—C24 and N2—C11—C8—C9 torsion angles are 10.0 (3)° and -170.6 (2)°, respectively. In the two independent molecules, the dihedral angles between the two aromatic rings are 64.0 (1)° and 66.5 (1) °, respectively. Bond lengths and angles are comparable to those observed for 4-chloro-*N*-[4-(dimethylamino)benzylidene]aniline (You *et al.*, 2004).

### Experimental

A mixture of 4-(diethylamino)benzaldehyde (0.01 mol) and 4-chloroaniline (0.01 mol) in ethanol (10 ml) was refluxed for 2 h. After cooling, filtration and drying, the title compound was obtained. The title compound (10 mg) was dissolved in ethanol (15 ml) and the solution was kept at room temperature for 5 d. Natural evaporation gave light-yellow single crystals of the title compound, suitable for X-ray analysis.

### Refinement

H atoms were initially located in a difference map and then refined in a riding model, with C–H = 0.93–0.97 Å and *U*_iso_(H) = 1.2*U*_eq_(C) and 1.5*U*_eq_(methyl C).

### Crystal data

**Table:**

C_17_H_19_ClN_2_	*F*(000) = 1216
*M**_r_* = 286.79	*D*_x_ = 1.221 Mg m^−^^3^
Monoclinic, *P*2_1_/*c*	Mo *K*α radiation, λ = 0.71073 Å
Hall symbol: -P 2ybc	Cell parameters from 1362 reflections
*a* = 20.153 (2) Å	θ = 2.4–21.4°
*b* = 8.7434 (7) Å	µ = 0.24 mm^−^^1^
*c* = 20.1446 (19) Å	*T* = 293 K
β = 118.444 (2)°	Block, light yellow
*V* = 3121.0 (5) Å^3^	0.25 × 0.22 × 0.18 mm
*Z* = 8	

### Data collection

**Table:**

Bruker SMART CCD area-detector diffractometer	5494 independent reflections
Radiation source: fine-focus sealed tube	4266 reflections with *I* > 2σ(*I*)
graphite	*R*_int_ = 0.034
ω scans	θ_max_ = 25.0°, θ_min_ = 3.0°
Absorption correction: multi-scan (*SADABS*; Sheldrick, 1996)	*h* = −23→23
*T*_min_ = 0.942, *T*_max_ = 0.958	*k* = −10→10
22416 measured reflections	*l* = −23→23

### Refinement

**Table:**

Refinement on *F*^2^	Primary atom site location: structure-invariant direct methods
Least-squares matrix: full	Secondary atom site location: difference Fourier map
*R*[*F*^2^ > 2σ(*F*^2^)] = 0.045	Hydrogen site location: inferred from neighbouring sites
*wR*(*F*^2^) = 0.127	H-atom parameters constrained
*S* = 1.07	*w* = 1/[σ^2^(*F*_o_^2^) + (0.0646*P*)^2^ + 0.3803*P*] where *P* = (*F*_o_^2^ + 2*F*_c_^2^)/3
5494 reflections	(Δ/σ)_max_ = 0.001
361 parameters	Δρ_max_ = 0.25 e Å^−^^3^
0 restraints	Δρ_min_ = −0.34 e Å^−^^3^

### Special details

**Table:**

Geometry. All e.s.d.'s (except the e.s.d. in the dihedral angle between two l.s. planes) are estimated using the full covariance matrix. The cell e.s.d.'s are taken into account individually in the estimation of e.s.d.'s in distances, angles and torsion angles; correlations between e.s.d.'s in cell parameters are only used when they are defined by crystal symmetry. An approximate (isotropic) treatment of cell e.s.d.'s is used for estimating e.s.d.'s involving l.s. planes.
Refinement. Refinement of *F*^2^ against ALL reflections. The weighted *R*-factor *wR* and goodness of fit *S* are based on *F*^2^, conventional *R*-factors *R* are based on *F*, with *F* set to zero for negative *F*^2^. The threshold expression of *F*^2^ > σ(*F*^2^) is used only for calculating *R*-factors(gt) *etc*. and is not relevant to the choice of reflections for refinement. *R*-factors based on *F*^2^ are statistically about twice as large as those based on *F*, and *R*- factors based on ALL data will be even larger.

### Fractional atomic coordinates and isotropic or equivalent isotropic displacement parameters (Å^2^)

**Table:**

	*x*	*y*	*z*	*U*_iso_*/*U*_eq_	
Cl2	0.85148 (4)	−0.53996 (7)	0.76082 (4)	0.0984 (2)	
Cl1	0.64613 (4)	1.53729 (7)	0.41511 (4)	0.1002 (2)	
N2	0.59641 (9)	1.07076 (17)	0.18309 (8)	0.0637 (4)	
N3	0.90381 (8)	0.57265 (16)	0.41592 (8)	0.0619 (4)	
C7	0.58844 (9)	0.83306 (19)	0.07682 (9)	0.0562 (4)	
H7A	0.5731	0.9299	0.0560	0.067*	
C5	0.60395 (9)	0.56348 (18)	0.05880 (9)	0.0530 (4)	
C23	0.91666 (10)	0.30238 (19)	0.44633 (10)	0.0581 (4)	
H23A	0.9321	0.2837	0.4103	0.070*	
C6	0.58355 (9)	0.71363 (19)	0.03069 (9)	0.0567 (4)	
H6A	0.5664	0.7319	−0.0204	0.068*	
N4	0.90566 (9)	−0.05533 (17)	0.58706 (9)	0.0659 (4)	
C24	0.91207 (10)	0.18314 (19)	0.48779 (9)	0.0583 (4)	
H24A	0.9252	0.0855	0.4798	0.070*	
C22	0.89856 (9)	0.45309 (18)	0.45679 (9)	0.0522 (4)	
C28	0.88144 (10)	0.0793 (2)	0.58554 (9)	0.0593 (4)	
H28A	0.8577	0.0992	0.6145	0.071*	
N1	0.59608 (9)	0.44244 (16)	0.01260 (9)	0.0627 (4)	
C11	0.62332 (9)	0.9374 (2)	0.20476 (10)	0.0568 (4)	
H11A	0.6495	0.9186	0.2564	0.068*	
C25	0.88817 (9)	0.20400 (19)	0.54188 (9)	0.0559 (4)	
C27	0.87455 (10)	0.47377 (19)	0.51171 (10)	0.0585 (4)	
H27A	0.8619	0.5712	0.5205	0.070*	
C8	0.61580 (9)	0.81317 (19)	0.15426 (9)	0.0539 (4)	
C10	0.63215 (10)	0.5445 (2)	0.13724 (10)	0.0612 (4)	
H10A	0.6474	0.4480	0.1585	0.073*	
C12	0.61080 (10)	1.18249 (19)	0.23937 (9)	0.0564 (4)	
C26	0.86968 (10)	0.3532 (2)	0.55194 (10)	0.0609 (4)	
H26A	0.8534	0.3708	0.5874	0.073*	
C29	0.89088 (10)	−0.16858 (19)	0.62846 (9)	0.0571 (4)	
C9	0.63739 (10)	0.6647 (2)	0.18232 (10)	0.0609 (4)	
H9A	0.6560	0.6476	0.2337	0.073*	
C15	0.63294 (11)	1.4018 (2)	0.34658 (11)	0.0645 (5)	
C34	0.95037 (10)	−0.25322 (19)	0.68199 (11)	0.0628 (5)	
H34A	0.9991	−0.2339	0.6903	0.075*	
C32	0.86616 (10)	−0.3967 (2)	0.70916 (10)	0.0625 (4)	
C17	0.68257 (10)	1.2175 (2)	0.29554 (11)	0.0669 (5)	
H17A	0.7238	1.1663	0.2972	0.080*	
C16	0.69412 (11)	1.3272 (2)	0.34924 (12)	0.0704 (5)	
H16A	0.7427	1.3504	0.3867	0.084*	
C14	0.56102 (11)	1.3704 (2)	0.29018 (12)	0.0730 (5)	
H14A	0.5199	1.4220	0.2885	0.088*	
C33	0.93858 (10)	−0.3652 (2)	0.72303 (11)	0.0660 (5)	
H33A	0.9792	−0.4194	0.7598	0.079*	
C2	0.61471 (11)	0.2868 (2)	0.04203 (12)	0.0709 (5)	
H2B	0.5867	0.2156	0.0012	0.085*	
H2C	0.5982	0.2725	0.0796	0.085*	
C13	0.55034 (10)	1.2624 (2)	0.23647 (11)	0.0674 (5)	
H13A	0.5019	1.2430	0.1977	0.081*	
C30	0.81858 (10)	−0.2037 (2)	0.61520 (11)	0.0696 (5)	
H30A	0.7778	−0.1496	0.5787	0.084*	
C4	0.57312 (12)	0.4657 (2)	−0.06713 (11)	0.0720 (5)	
H4B	0.5317	0.5382	−0.0878	0.086*	
H4C	0.5546	0.3696	−0.0936	0.086*	
C19	0.88835 (11)	0.72995 (19)	0.42921 (12)	0.0699 (5)	
H19A	0.9164	0.7989	0.4141	0.084*	
H19B	0.9068	0.7438	0.4829	0.084*	
C21	0.92008 (12)	0.5464 (2)	0.35369 (12)	0.0730 (5)	
H21A	0.9627	0.4772	0.3705	0.088*	
H21B	0.9347	0.6427	0.3405	0.088*	
C31	0.80608 (11)	−0.3174 (2)	0.65510 (12)	0.0714 (5)	
H31A	0.7572	−0.3402	0.6455	0.086*	
C20	0.85481 (14)	0.4803 (3)	0.28411 (12)	0.0883 (7)	
H20A	0.8693	0.4669	0.2455	0.132*	
H20B	0.8126	0.5488	0.2664	0.132*	
H20C	0.8410	0.3832	0.2962	0.132*	
C3	0.63511 (14)	0.5236 (3)	−0.08195 (13)	0.0882 (7)	
H3A	0.6160	0.5365	−0.1353	0.132*	
H3B	0.6758	0.4512	−0.0630	0.132*	
H3C	0.6531	0.6201	−0.0570	0.132*	
C18	0.80592 (12)	0.7741 (2)	0.38778 (15)	0.0933 (7)	
H18A	0.8003	0.8782	0.3995	0.140*	
H18B	0.7776	0.7081	0.4031	0.140*	
H18C	0.7874	0.7642	0.3344	0.140*	
C1	0.69744 (13)	0.2482 (3)	0.07706 (15)	0.0991 (8)	
H1A	0.7053	0.1449	0.0954	0.149*	
H1B	0.7257	0.3167	0.1182	0.149*	
H1C	0.7141	0.2584	0.0398	0.149*	

### Atomic displacement parameters (Å^2^)

**Table:**

	*U*^11^	*U*^22^	*U*^33^	*U*^12^	*U*^13^	*U*^23^
Cl2	0.1067 (5)	0.0772 (4)	0.1268 (5)	−0.0034 (3)	0.0682 (4)	0.0204 (3)
Cl1	0.1127 (5)	0.0755 (4)	0.1135 (5)	−0.0022 (3)	0.0549 (4)	−0.0307 (3)
N2	0.0695 (9)	0.0627 (9)	0.0590 (9)	0.0001 (7)	0.0307 (8)	0.0018 (7)
N3	0.0668 (9)	0.0526 (8)	0.0632 (9)	−0.0015 (6)	0.0283 (8)	−0.0032 (7)
C7	0.0584 (10)	0.0542 (9)	0.0571 (10)	0.0021 (7)	0.0285 (8)	0.0076 (7)
C5	0.0499 (9)	0.0542 (9)	0.0561 (9)	−0.0034 (7)	0.0263 (8)	0.0027 (7)
C23	0.0627 (10)	0.0593 (10)	0.0541 (9)	0.0065 (8)	0.0292 (8)	−0.0027 (8)
C6	0.0590 (10)	0.0612 (10)	0.0489 (9)	−0.0001 (7)	0.0250 (8)	0.0058 (8)
N4	0.0775 (10)	0.0637 (9)	0.0628 (9)	0.0058 (7)	0.0386 (8)	−0.0002 (7)
C24	0.0643 (10)	0.0539 (9)	0.0556 (10)	0.0077 (7)	0.0279 (8)	−0.0038 (7)
C22	0.0468 (8)	0.0536 (9)	0.0462 (9)	−0.0011 (7)	0.0140 (7)	−0.0058 (7)
C28	0.0617 (10)	0.0649 (11)	0.0503 (9)	0.0030 (8)	0.0258 (8)	−0.0044 (8)
N1	0.0700 (9)	0.0542 (8)	0.0627 (9)	−0.0029 (6)	0.0306 (8)	−0.0007 (7)
C11	0.0531 (9)	0.0658 (11)	0.0538 (10)	−0.0010 (8)	0.0275 (8)	0.0034 (8)
C25	0.0561 (9)	0.0593 (10)	0.0469 (9)	0.0017 (7)	0.0202 (8)	−0.0042 (7)
C27	0.0618 (10)	0.0533 (9)	0.0547 (10)	0.0029 (7)	0.0231 (8)	−0.0117 (8)
C8	0.0506 (9)	0.0603 (10)	0.0535 (9)	−0.0019 (7)	0.0269 (8)	0.0015 (7)
C10	0.0674 (11)	0.0572 (10)	0.0583 (10)	0.0029 (8)	0.0295 (9)	0.0111 (8)
C12	0.0627 (10)	0.0536 (9)	0.0563 (10)	−0.0012 (7)	0.0311 (8)	0.0073 (7)
C26	0.0655 (10)	0.0649 (11)	0.0511 (9)	0.0028 (8)	0.0268 (8)	−0.0111 (8)
C29	0.0644 (10)	0.0549 (9)	0.0543 (9)	0.0026 (8)	0.0302 (8)	−0.0080 (7)
C9	0.0656 (10)	0.0670 (11)	0.0509 (9)	0.0011 (8)	0.0285 (8)	0.0089 (8)
C15	0.0738 (12)	0.0501 (9)	0.0743 (12)	−0.0026 (8)	0.0390 (10)	−0.0017 (8)
C34	0.0560 (10)	0.0571 (10)	0.0753 (12)	0.0021 (8)	0.0313 (9)	−0.0027 (9)
C32	0.0703 (11)	0.0512 (9)	0.0708 (11)	−0.0025 (8)	0.0376 (10)	−0.0052 (8)
C17	0.0571 (10)	0.0704 (11)	0.0779 (12)	0.0002 (8)	0.0358 (10)	−0.0042 (10)
C16	0.0578 (10)	0.0689 (12)	0.0783 (13)	−0.0053 (8)	0.0275 (10)	−0.0101 (10)
C14	0.0632 (11)	0.0575 (11)	0.0990 (15)	0.0086 (8)	0.0391 (11)	0.0011 (10)
C33	0.0603 (10)	0.0568 (10)	0.0727 (12)	0.0047 (8)	0.0250 (9)	0.0030 (9)
C2	0.0719 (12)	0.0535 (10)	0.0827 (13)	−0.0025 (8)	0.0332 (10)	0.0006 (9)
C13	0.0558 (10)	0.0578 (10)	0.0772 (12)	0.0040 (8)	0.0225 (9)	0.0035 (9)
C30	0.0580 (11)	0.0717 (12)	0.0693 (12)	0.0081 (9)	0.0223 (9)	0.0018 (9)
C4	0.0848 (13)	0.0649 (11)	0.0613 (11)	−0.0090 (9)	0.0307 (10)	−0.0107 (9)
C19	0.0666 (11)	0.0515 (10)	0.0813 (13)	−0.0012 (8)	0.0267 (10)	−0.0028 (9)
C21	0.0829 (13)	0.0669 (12)	0.0791 (13)	−0.0034 (9)	0.0465 (11)	0.0061 (10)
C31	0.0574 (10)	0.0690 (12)	0.0881 (14)	−0.0030 (9)	0.0349 (10)	−0.0062 (10)
C20	0.1086 (18)	0.0907 (15)	0.0606 (12)	0.0092 (12)	0.0362 (12)	0.0058 (11)
C3	0.1165 (19)	0.0832 (14)	0.0860 (16)	0.0070 (13)	0.0652 (15)	−0.0008 (12)
C18	0.0720 (13)	0.0778 (14)	0.1146 (18)	0.0135 (10)	0.0319 (13)	0.0052 (13)
C1	0.0780 (15)	0.0875 (16)	0.1166 (19)	0.0177 (12)	0.0342 (14)	0.0075 (14)

### Geometric parameters (Å, °)

**Table:**

Cl2—C32	1.7415 (19)	C15—C16	1.373 (3)
Cl1—C15	1.7407 (19)	C15—C14	1.378 (3)
N2—C11	1.273 (2)	C34—C33	1.373 (2)
N2—C12	1.418 (2)	C34—H34A	0.93
N3—C22	1.366 (2)	C32—C31	1.370 (3)
N3—C21	1.457 (2)	C32—C33	1.377 (2)
N3—C19	1.463 (2)	C17—C16	1.380 (3)
C7—C6	1.370 (2)	C17—H17A	0.93
C7—C8	1.396 (2)	C16—H16A	0.93
C7—H7A	0.93	C14—C13	1.373 (3)
C5—N1	1.368 (2)	C14—H14A	0.93
C5—C6	1.411 (2)	C33—H33A	0.93
C5—C10	1.411 (2)	C2—C1	1.507 (3)
C23—C24	1.366 (2)	C2—H2B	0.97
C23—C22	1.410 (2)	C2—H2C	0.97
C23—H23A	0.93	C13—H13A	0.93
C6—H6A	0.93	C30—C31	1.375 (3)
N4—C28	1.269 (2)	C30—H30A	0.93
N4—C29	1.415 (2)	C4—C3	1.504 (3)
C24—C25	1.397 (2)	C4—H4B	0.97
C24—H24A	0.93	C4—H4C	0.97
C22—C27	1.414 (2)	C19—C18	1.511 (3)
C28—C25	1.447 (2)	C19—H19A	0.97
C28—H28A	0.93	C19—H19B	0.97
N1—C4	1.459 (2)	C21—C20	1.507 (3)
N1—C2	1.460 (2)	C21—H21A	0.97
C11—C8	1.446 (2)	C21—H21B	0.97
C11—H11A	0.93	C31—H31A	0.93
C25—C26	1.398 (2)	C20—H20A	0.96
C27—C26	1.361 (2)	C20—H20B	0.96
C27—H27A	0.93	C20—H20C	0.96
C8—C9	1.400 (2)	C3—H3A	0.96
C10—C9	1.360 (2)	C3—H3B	0.96
C10—H10A	0.93	C3—H3C	0.96
C12—C17	1.381 (3)	C18—H18A	0.96
C12—C13	1.381 (2)	C18—H18B	0.96
C26—H26A	0.93	C18—H18C	0.96
C29—C34	1.384 (2)	C1—H1A	0.96
C29—C30	1.385 (3)	C1—H1B	0.96
C9—H9A	0.93	C1—H1C	0.96
			
C11—N2—C12	117.75 (15)	C12—C17—H17A	119.5
C22—N3—C21	120.79 (14)	C15—C16—C17	119.11 (18)
C22—N3—C19	121.66 (16)	C15—C16—H16A	120.4
C21—N3—C19	117.42 (16)	C17—C16—H16A	120.4
C6—C7—C8	121.71 (15)	C13—C14—C15	119.69 (17)
C6—C7—H7A	119.1	C13—C14—H14A	120.2
C8—C7—H7A	119.1	C15—C14—H14A	120.2
N1—C5—C6	121.97 (15)	C34—C33—C32	119.40 (17)
N1—C5—C10	121.69 (15)	C34—C33—H33A	120.3
C6—C5—C10	116.33 (15)	C32—C33—H33A	120.3
C24—C23—C22	121.62 (16)	N1—C2—C1	114.34 (17)
C24—C23—H23A	119.2	N1—C2—H2B	108.7
C22—C23—H23A	119.2	C1—C2—H2B	108.7
C7—C6—C5	121.59 (15)	N1—C2—H2C	108.7
C7—C6—H6A	119.2	C1—C2—H2C	108.7
C5—C6—H6A	119.2	H2B—C2—H2C	107.6
C28—N4—C29	118.48 (15)	C14—C13—C12	120.69 (17)
C23—C24—C25	121.73 (15)	C14—C13—H13A	119.7
C23—C24—H24A	119.1	C12—C13—H13A	119.7
C25—C24—H24A	119.1	C31—C30—C29	121.10 (17)
N3—C22—C23	121.71 (16)	C31—C30—H30A	119.4
N3—C22—C27	121.83 (15)	C29—C30—H30A	119.5
C23—C22—C27	116.47 (16)	N1—C4—C3	114.07 (18)
N4—C28—C25	124.55 (17)	N1—C4—H4B	108.7
N4—C28—H28A	117.7	C3—C4—H4B	108.7
C25—C28—H28A	117.7	N1—C4—H4C	108.7
C5—N1—C4	120.95 (14)	C3—C4—H4C	108.7
C5—N1—C2	121.38 (15)	H4B—C4—H4C	107.6
C4—N1—C2	117.57 (15)	N3—C19—C18	114.41 (16)
N2—C11—C8	124.21 (16)	N3—C19—H19A	108.7
N2—C11—H11A	117.9	C18—C19—H19A	108.7
C8—C11—H11A	117.9	N3—C19—H19B	108.7
C24—C25—C26	116.71 (16)	C18—C19—H19B	108.7
C24—C25—C28	122.85 (15)	H19A—C19—H19B	107.6
C26—C25—C28	120.43 (16)	N3—C21—C20	113.96 (18)
C26—C27—C22	121.02 (15)	N3—C21—H21A	108.8
C26—C27—H27A	119.5	C20—C21—H21A	108.8
C22—C27—H27A	119.5	N3—C21—H21B	108.8
C7—C8—C9	116.62 (16)	C20—C21—H21B	108.8
C7—C8—C11	123.10 (15)	H21A—C21—H21B	107.7
C9—C8—C11	120.27 (15)	C32—C31—C30	119.47 (18)
C9—C10—C5	121.25 (16)	C32—C31—H31A	120.3
C9—C10—H10A	119.4	C30—C31—H31A	120.3
C5—C10—H10A	119.4	C21—C20—H20A	109.5
C17—C12—C13	118.74 (17)	C21—C20—H20B	109.5
C17—C12—N2	122.91 (16)	H20A—C20—H20B	109.5
C13—C12—N2	118.32 (16)	C21—C20—H20C	109.5
C27—C26—C25	122.45 (17)	H20A—C20—H20C	109.5
C27—C26—H26A	118.8	H20B—C20—H20C	109.5
C25—C26—H26A	118.8	C4—C3—H3A	109.5
C34—C29—C30	118.21 (17)	C4—C3—H3B	109.5
C34—C29—N4	119.08 (16)	H3A—C3—H3B	109.5
C30—C29—N4	122.63 (16)	C4—C3—H3C	109.5
C10—C9—C8	122.46 (16)	H3A—C3—H3C	109.5
C10—C9—H9A	118.8	H3B—C3—H3C	109.5
C8—C9—H9A	118.8	C19—C18—H18A	109.5
C16—C15—C14	120.64 (18)	C19—C18—H18B	109.5
C16—C15—Cl1	119.81 (15)	H18A—C18—H18B	109.5
C14—C15—Cl1	119.54 (15)	C19—C18—H18C	109.5
C33—C34—C29	121.09 (17)	H18A—C18—H18C	109.5
C33—C34—H34A	119.5	H18B—C18—H18C	109.5
C29—C34—H34A	119.5	C2—C1—H1A	109.5
C31—C32—C33	120.69 (18)	C2—C1—H1B	109.5
C31—C32—Cl2	120.11 (15)	H1A—C1—H1B	109.5
C33—C32—Cl2	119.20 (14)	C2—C1—H1C	109.5
C16—C17—C12	121.07 (17)	H1A—C1—H1C	109.5
C16—C17—H17A	119.5	H1B—C1—H1C	109.5
